# Reflex Neuroses Due to Constipation

**Published:** 1888-05

**Authors:** 


REFLEX NEUROSES DUE TO CONSTIPATION.—Besides various circulatory disorders, such as palpitation, irregular pulse and vertigo, which may be attributed to excitation of the cardiac branches of the great sympathetic, Kish maintains that many obstinate cases of migraine are due to constipation. Some forms of abdominal neuralgia, lumbo-sacral pains, sciatica ovarian pains and neuralgia of the fifth pair are often due to habitual constipation. Kish has collected forty-eight cases of neuralgia cured by relieving constipation.—*Journal des Sciences Med.*

